# Effects of bevacizumab administration on the hypoxia - induced pulmonary hypertension rat model

**DOI:** 10.3906/sag-2101-76

**Published:** 2021-10-21

**Authors:** Canan DEMİR, Meral KARAMAN, Eyüp Sabri UÇAN, Ali Necati GÖKMEN, Duygu GÜREL, Şadiye Canan ÇOKER, Yasemen ADALI, Osman YILMAZ

**Affiliations:** 1 Occupational Diseases Clinic, Occupational and Environmental Diseases Hospital, Ankara Turkey; 2 Department of Medical Microbiology, Faculty of Medicine, Dokuz Eylül University, İzmir Turkey; 3 Department of Chest Diseases, Faculty of Medicine, Dokuz Eylül University, İzmir Turkey; 4 Department of Anesthesiology and Reanimation, Faculty of Medicine, Dokuz Eylül University, İzmir Turkey; 5 Department of Medical Pathology, Faculty of Medicine, Dokuz Eylül University, İzmir Turkey; 6 Department of Medical Biochemistry, Faculty of Medicine, Dokuz Eylül University, İzmir Turkey; 7 Department of Medical Pathology, Faculty of Medicine, Izmir University of Economics, İzmir Turkey; 8 Department of Laboratory Animal Science, Faculty of Medicine, Dokuz Eylül University, İzmir Turkey

**Keywords:** Hypertension, pulmonary, bevacizumab, hypoxia

## Abstract

**Background/aim:**

Bevacizumab is a chemotherapeutic drug, which selectively binds to vascular endothelial growth factor (VEGF) and mainly inhibits angiogenesis and neovascularization. We aimed to study the possible effects of bevacizumab on right ventricular pressure (RVP), right ventricular hypertrophy, and VEGF, in hypoxia - induced pulmonary hypertension (PH) rat model.

**Materials and methods:**

24 adult Wistar Albino rats were randomly divided into four groups: control group - saline; Bevacizumab Group; PH Group; PH + Bevacizumab Group. In hypoxia - induced model, 10% oxygen and 90% nitrogen were applied in a plexiglas box for eight days to PH Group and PH + Bevacizumab Group. On day eight, RVPs were measured directly from the heart, and then animals were sacrificed. Heart and lung tissues were examined, and Fulton index was measured.

**Results:**

RVP, Fulton index, and tissue VEGF scores were significantly lower in PH + Bevacizumab group than PH group: median (ranges), RVP, mmHg, 37.8 (33.0–39.0) and 32.3 (28.0–35.0), p: 0.01; Fulton index: 0.30 (0.29–0.33) and 0.25 (0.24–0.26), p: 0.003; tissue VEGF scores: 5.1 (4.8–5.3) and 4.0 (3.8 4.1), p: 0.004, respectively.

**Conclusion:**

Bevacizumab, which is indeed an antineoplastic agent, might have a favorable effect on hypoxia - induced pulmonary hypertension.

## 1. Introduction

Pulmonary hypertension (PH) is a clinical disorder that may complicate cardiovascular and respiratory diseases [1]. PH is defined as an increase in mean pulmonary arterial pressure (PAPm) ⩾ 25 mmHg at rest measured during right heart catheterization [2] in humans. 

The most important mechanisms in PH pathogenesis revealed by scientific studies are as follows: 1. Endothelial dysfunction leading to vasoconstriction and thrombosis, 2. Genetic mutations such as BMPR - 2 and EIF2AK4, 3. Cancer - like cellular responses (increased proliferation, impaired apoptosis and glycolytic metabolism in pulmonary artery smooth muscle, fibroblasts, and endothelial cells), 4. Rho - kinase activation and refractory vasoconstriction, 5. Right ventricular (RV) failure (as a result of increased afterload) [3].

Vascular endothelial growth factor (VEGF) is an endothelial cell mitogen and angiogenic factor that binds to endothelial cells via tyrosine kinase receptors (two subtypes: VEGFR – 1 / KDR and VEGFR – 2 / Flt) in the pulmonary circulation, which could be related to cancer - like cellular responses. Both VEGF and its receptors are upregulated in acute and chronic hypoxia [4]. VEGF expression, VEGFR - 1 levels in pulmonary endothelium, and VEGFR - 2 levels in plexiform lesions are also increased [5,6]. In rat models, chronic VEGFR - 2 blockade together with hypoxia may induce pulmonary endothelial cell apoptosis. In some apoptosis - resistant cell groups, an increased proliferation may occur, which can cause a severe form of PH [7]. 

Chronic hypoxia is one of the frequent causes of PH. Its effects on the pulmonary vascular system have been extensively studied in both in vitro and in vivo experimental studies [8-10]. Acute hypoxia causes reversible vasoconstriction in pulmonary arteries by increasing endothelin - 1 (ET - 1), serotonin, hypoxia, and redox - sensitive potassium channel activity in pulmonary vascular smooth muscle cells [10]. Chronic hypoxia, on the other hand, often causes irreversible effects and vascular remodelling. However, the histopathological changes in the chronic hypoxia animal model of PH are not totally irreversible [11]. Despite these differences, the chronic hypoxia animal model is frequently used in PH studies due to its ease of application and the fact that it causes a significant PH in animals.

Bevacizumab is a drug used in the treatment of some cancers, mainly colon cancer. It is a recombinant humanized monoclonal IgG1 antibody that selectively binds to VEGF and neutralizes this mediator’s biological activity. It mainly inhibits angiogenesis and neovascularisation and thus, effectively controls cancer cells’ proliferation and prevents metastases [12].

Due to VEGF’s inhibitory effect on angiogenesis, we hypothesized that bevacizumab might affect PH pathogenesis. Thus, we aimed to study the possible effects of bevacizumab in hypoxia - induced PH animal model.

## 2. Materials and methods

### 2.1. Experimental design 

Ethical approval for all the experimental procedures was obtained from the Dokuz Eylül University Local Ethics Committee for Animal Experiments (392009, İzmir, Turkey). 24 adult male Wistar Albino rats between 200–250 mg were used in this study. Rats were randomly divided into four groups, with six rats in each group. During the experiment, they were housed in standard rat cages and fed with standard rodent food. Relative humidity was measured as 55% in the accommodation chambers. The body weights of all rats included in the study were weighed and recorded when starting the experiment. All animals were fed ad libitum and exposed to the same day-night cycle (circadian rhythm: 12 h dark, 12 h light). Experimental groups were planned as: Group 1 (control group - saline): As of day 0, rats breathe room air, and 0.9% saline was applied intraperitoneally (i.p.) on day 5. Group 2 (Bevacizumab Group): As of day 0, rats breathe room air, and 1 mg / kg bevacizumab was applied i.p. on day 5. In the literature, doses of bevacizumab applied in animal (rats) studies mainly ranged between 0.1 mg / kg and 10 mg / kg [13,14]. We chose the dose of 1 mg / kg as an average of this range. Group 3 (PH group): As of day 0, rats breathe a mixture of 10% oxygen and 90% nitrogen in a closed chamber, and 0.9% saline was applied i.p. on day 5. Group 4 (PH + Bevacizumab group): As of day 0, rats breathe a mixture of 10% oxygen and 90% nitrogen in a closed chamber, and 1 mg / kg bevacizumab was applied i.p. on day 5. Animals in all groups were sacrificed on day 8 after the necessary measurements were made. The duration of follow up was determined as 8 days based on our observations in the pilot study. Table summarizes the experiment groups. Hypoxia-induced model used in the experiments is a normobaric model and was carried out by applying a gas mixture consisting of 10% oxygen and 90% nitrogen at a rate of 2 lt / min to a Plexiglas box with dimensions of 110 × 56 × 35 cm and a total volume of 215 lt, for 23 h a day for eight days [15-20]. The Plexiglas box was opened twice a day to clean the cages and for feeding purposes, each lasting approximately 30 min [16–19]. The levels of oxygen, carbon dioxide, and nitrogen in the Plexiglas box were measured and recorded three times a day with an anaesthetic gas monitor (Anaesthetic Gas Monitor Type 1304, Bruel & Kjaer). Necessary adjustments have been applied to keep the oxygen level between 10%–15% and the carbon dioxide level < 0.2% in the box environment. The average room temperature was between 22–24 °C. Soda-lime was sprinkled on the box’s basement to bind the box environment’s excess carbon dioxide and humidity [16,17,19,20]. Soda-lime material was changed daily.

**Table:** Description of the experiment groups.

Group	Description
Group 1 (Control Group - Saline)	Breathe room air, 0.9% saline i.p. applied on day 5
Group 2 (Bevacizumab Group)	Breathe room air, 1 mg / kg bevacizumab i.p. applied on day 5
Group 3 (PH group)	Breathe a mixture of 10% oxygen and 90% nitrogen in a closed chamber, 0.9% saline i.p. applied on day 5
Group 4 (PH + Bevacizumab group)	Breathe a mixture of 10% oxygen and 90% nitrogen in a closed chamber, 1 mg / kg bevacizumab i.p. applied on day 5

### 2.2. Hemodynamic measurements

The animals were weighed, and their breathing patterns, oral intakes, and heart rates were monitored daily. On Day 5, 0.9% saline i.p. was applied to Group 1 and 3, and 1 mg / kg bevacizumab i.p. was applied to Group 2 and 4. On the eighth day, all rats were anesthetized with a combination of ketamine and xylazine (5 mg / kg xylazine i.p. and 35 mg / kg ketamine i.p.). Rats were fixed in the supine position. Thoracic cavity was opened in all rats by thoracotomy and right ventricular pressures (RVP) were measured directly from the heart as described before [21]. Briefly, right ventricular mean pressure (reflecting pulmonary artery pressure) and left ventricular systolic pressure (reflecting systemic arterial pressure) were recorded by using 20 G catheters with pressure transducers continuously. Pressure measurements were made from the right ventricle instead of the pulmonary artery because pulmonary artery catheterization is technically very difficult in rats with pulmonary hypertension due to high pressures. Petaş KMA 250 Pressure Transducer and Recording System was used for measurements. After the pressure measurements were completed, the animals were sacrificed by taking their whole - body blood (approximately 5 mL for each animal) via an intracardiac puncture, and the heart and lungs of animals were dissected for further examinations.

### 2.3. Serum VEGF measurements

VEGF levels were measured in the serum by using Invitrogen (CA_USA) Rt VEGF kit and solid-phase sandwich Enzyme Linked - Immuno Sorbent Assay (ELISA) method.

### 2.4. Morphometrical and pathological evaluation

Fulton Index (Right Ventricle / Left Ventricle + Septum Weight Ratio) [22] was measured on heart tissues. Lungs and hearts were fixed in 10% neutral buffered formalin for at least 24 h. The fixed heart and lung tissues were processed in the pathology laboratory within 24 h. Tissue samples were routinely processed, embedded in paraffin, and cut to 5µ sections. The histological sections were stained with hematoxylin & eosin (H & E). In the H & E - stained sections, the thickness of the muscularis mucosa layer (Medial Thickness = MT) of 25 small pulmonary arteries (diameter < 100 µm) was measured morphometrically at × 40 magnification. Besides, the external diameter of each vessel was measured [15,17,23,24]. Then, medial thickness / external diameter (distance between external elastic laminae) ratios were calculated, and each value was multiplied by 100 to convert these values to percentage values. Thus, medial thickness% (MT%) values were obtained. 

### 2.5. Immunohistochemistry

Sections from paraffin - embedded tissue blocks were taken on lysine - coated slides. After deparaffinization and rehydration, slides were placed in a plastic jar filled with phosphate - buffered saline (PBS, pH: 7.2). Endogenous peroxidase activity was quenched with hydrogen peroxide. The tissue sections were then incubated with polyclonal antibody to VEGF (dilution: 1 / 200, ab46154_Abcam_MA_USA). Primary antibodies were applied overnight at 4 °C and washed in PBS. The binding of VEGF was detected using the streptavidin - biotin immunoperoxidase method. Diaminobenzidine was used as a chromogen. In VEGF antibody - stained sections, 25 pulmonary arteries were examined randomly at × 40 magnification, and VEGF tissue score was used as staining criteria. Staining intensity and staining percentages of each pulmonary artery endothelium with VEGF antibody were used to obtain this score. Staining intensity was scored as in the following: No staining = 0, weak intensity of staining = 1, medium intensity of staining = 2, strong intensity of staining = 3. Staining percentage was scored as: < 10% staining = 1, 10 - 50% staining = 2, 50 - 90% staining = 3 and 100% staining = 4. VEGF tissue score of each vessel was calculated by summing the staining intensity score and the staining percentage scores [23,25]. Two pathologists performed all these pathological procedures who did not know which animal and which experimental group the sections belonged to (blind study technique). Afterward, the average of the morphometric measurements (MT and MT%) and immunohistochemistry measurements (tissue VEGF scores) of each animal were calculated, and these values were used in statistical analysis.

### 2.6. Statistical analysis

Measurements of weight, RVP, Fulton İndex, MT, and tissue VEGF scores were presented in the Results Section as median and ranges and in the figures as mean and standard deviations (SD). We applied hierarchical comparisons based on hypoxia - induced PH modelling as our primary hypothesis (between PH group and PH + Bevacizumab group). Pair - wise comparisons in the non-PH and PH groups included control group - saline vs. Bevacizumab group, and PH group vs. PH + Bevacizumab group, respectively [26]. Accordingly, p value for pair - wise comparisons was corrected by dividing 0.05 to two, i.e. 0.025. For intergroup comparisons, Kruskal–Wallis test followed by Mann–Whitney test with Bonferroni correction was used. Friedman S test was used for intragroup comparisons, and Wilcoxon test with Bonferroni correction was used for pairwise comparisons. For weight changes, intragroup comparisons were made between D0 to D5, and D5 to D8. P values less than 0.05, in the two - sided testing were accepted for statistical significance. P values to define the statistical significance for pair - wise comparisons, as outlined above were depicted in the figure legends. SPSS, version 18.0 (PASW Statistics, USA) was used in the statistical analysis.

## 3. Results

### 3. 1. Weight measurements

**Figure 1:**
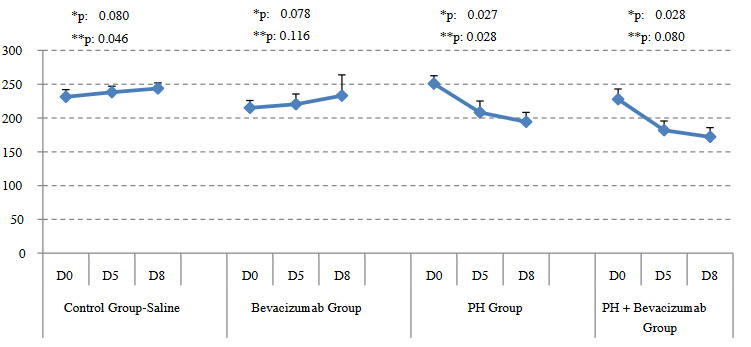
Weight measurements of the experiment groups, in day 0, day 5, and day 8 (d0, d5, and d8, respectively): Weight changes were significant in the control group - saline from d5 to d8, p: 0.046; in the ph group from d0 to d5, p: 0.027, and from d5 to d8, p: 0.028; and in the PH + Bevacizumab group from d0 to d5, p: 0.028. Mean and SD of the measurements were provided in the figure. *: Weight change from d0 to d5, **: weight change from D5 to D8.

Weight measurements of the groups from Day 0 to Day 5 and Day 5 to Day 8, were (median, and ranges) in grams were as follows: control group - saline: 236, 216 to 240 – 239, 228 to 250 – 246, 228 to 250; Bevacizumab group: 216, 200 to 230 – 221, 204 to 245 – 230, 196 to 280; PH group: 253, 230 to 264 – 213, 176 to 225 – 200, 167 to 204; PH + Bevacizumab group: 226, 209 to 245 – 184, 158 to 195 – 165, 158 to 190, respectively. Weight changes were significant in the control group - saline from day 5 to day 8, p: 0.046; in the PH group from day 0 to day 5, p: 0.027, and from day 5 to day 8, p: 0.028; and in the PH + Bevacizumab group from day 0 to day 5, p: 0.028. Mean, and SD of the weight measurements of the experiment groups, in day 0, day 5, and day 8 were provided in Figure 1. 

### 3.2. Right ventricular pressure (RVP)

**Figure 2:**
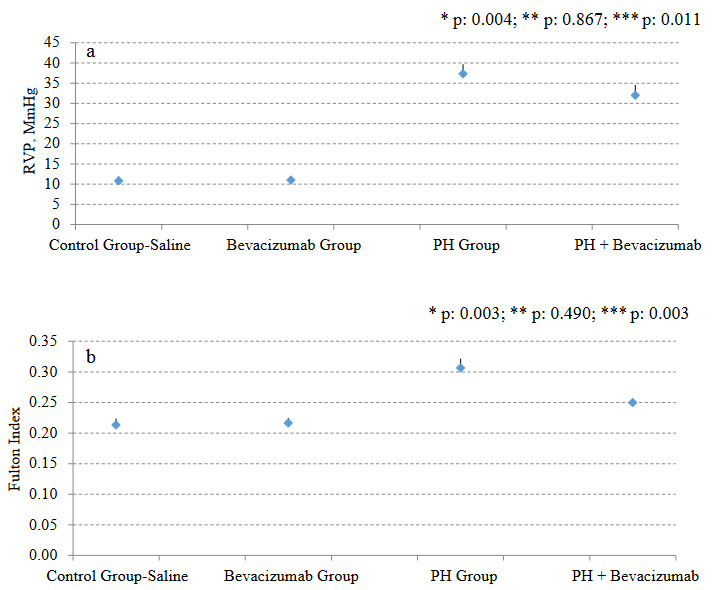
a. RVP measurements of the experiment groups; b. Fulton Index measurements of the experiment groups. Mean, and SD of the measurements were provided in the figures. P value to define statistical significance for comparisons of control group - saline vs bevacizumab group, and PH group vs PH + Bevacizumab group was 0.025. *: control group - saline vs PH group; **: control group - saline vs Bevacizumab group; ***: PH group vs PH + Bevacizumab group.

RVP was significantly greater in the PH group than control group - saline (p: 0.004), and PH + Bevacizumab group (p: 0.01). Median and ranges in mmHg were control group - saline: 11.0, 9.0 to 12.0; Bevacizumab group: 11.0, 10.0 to 12.0; PH group: 37.8, 33.0 to 39.0; PH + Bevacizumab group: 32.3, 28.0 to 35.0; p < 0.001 for all groups’ comparison. RVP measurements of the experiment groups are shown in Figure 2a. 

### 3.3. Fulton index 

Fulton index was significantly greater in the PH Group than control group - saline (p: 0.003), and PH + Bevacizumab group (p: 0.003). Median and ranges were control group - saline: 0.21, 0.20 to 0.23; Bevacizumab group: 0.21, 0.20 to 0.23; PH group: 0.30, 0.29 to 0.33; PH + Bevacizumab group: 0.25, 0.24 to 0.26; p < 0.001 for all groups’ comparison. Fulton index measurements of the experiment groups are shown in Figure 2b.

### 3.4. Serum VEGF levels

**Figure 3:**
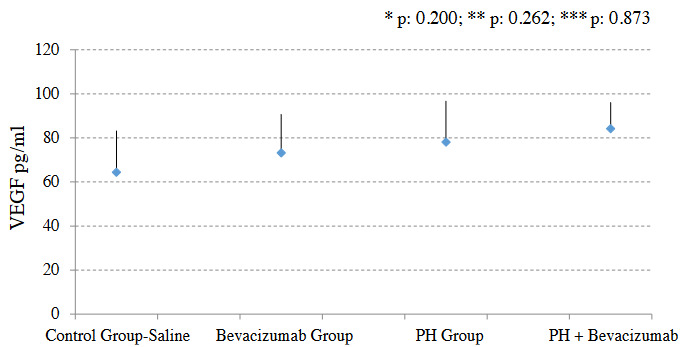
Serum VEGF levels of the experiment groups. Mean, and SD of the measurements were provided in the figure. P value to define statistical significance for control group - saline vs bevacizumab group, and PH group vs PH + Bevaciuzmab group was 0.025. *: control group - saline vs PH group; **: control group - saline vs bevacizumab group; ***: PH group vs. PH + Bevaciuzmab group.

Serum VEGF level was not significantly different between the groups. Median and ranges in pg / mL were control group - saline: 56.9, 41.5 to 96.4; Bevacizumab Group: 65.5, 55.2 to 103.8; PH Group: 89.5, 52.8 to 134.2; PH + Bevacizumab Group: 83.9, 70.2 to 97.2; p: 0.14 for all groups’ comparison. Serum VEGF levels of the experiment groups are shown in Figure 3. 

### 3.5. Medial thickness (MT)

**Figure 4:**
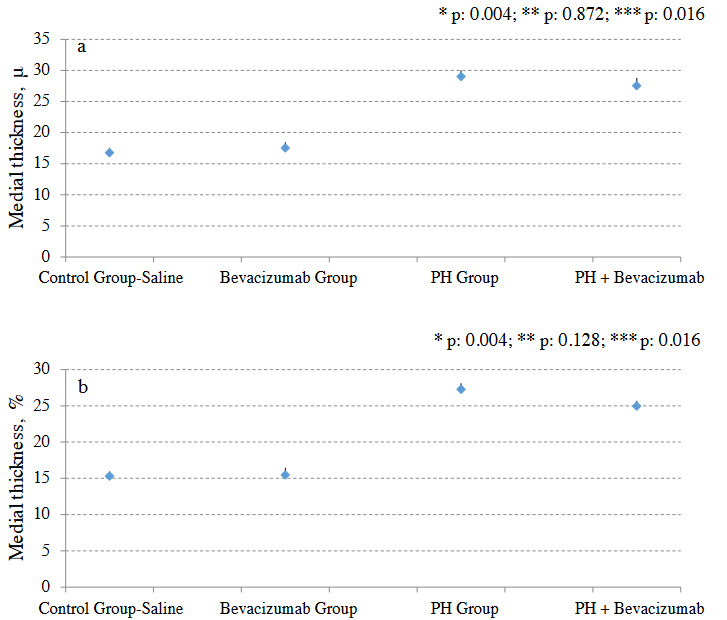
a. Muscularis mucosa layer thickness (medial thickness: MT) of the experiment groups; b. MT% of the experiment groups. Mean, and SD of the measurements were provided in the figure. P value to define statistical significance for comparisons of control group - saline vs bevacizumab group, and ph group vs ph + bevacizumab Group was 0.025. *: control group - saline vs PH group; **: control group - saline vs bevacizumab group; ***: PH Group vs. PH + bevacizumab group.

**Figure 5:**
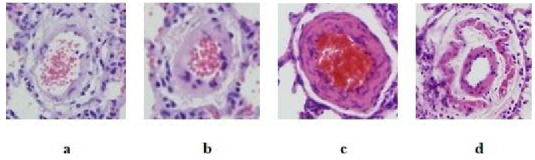
Hematoxylin & Eosin staining of small pulmonary arteries (at × 40 magnification) a. Normal pulmonary artery; b. Pulmonary artery with moderate thickening of the muscular layer; c. Pulmonary artery with significant thickening of the muscular layer; d. Pulmonary artery with a double muscular layer.

MT was significantly greater in the PH Group than control group - saline (p: 0.004), and PH + Bevacizumab Group (p: 0. 01). Median and ranges in µm were control group - saline: 15.5, 14.3 to 16.5; Bevacizumab Group: 15.3, 14.6 to 17.1; PH Group: 27.4, 26.0 to 28.3; PH + Bevacizumab Group: 24.4, 24.1 to 27.2; p < 0.001 for all groups’ comparison. MT and MT% of the experiment groups are shown in Figures 4a and 4b. Four different H & E - stained sections of small pulmonary arteries are shown in Figures 5a–5d.

### 3.6. VEGF tissue score

**Figure 6:**
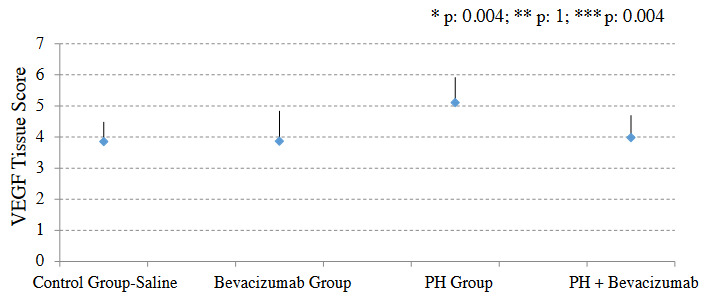
VEGF tissue scores of the experiment groups. Mean, and SD of the measurements are provided in the figure. P value to define statistical significance for comparisons of control group - saline vs bevacizumab group, and PH Group vs PH + bevacizumab group was 0.025. *: control group - saline vs. PH group; **: control group - saline vs bevacizumab group; ***: PH group vs PH + bevacizumab group.

**Figure 7:**
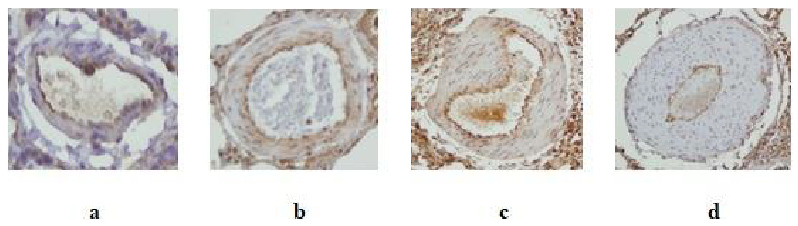
VEGF antibody staining of small pulmonary arteries (with 1 / 200 dilution and × 40 magnification) a. Partial and mild intensity staining of a normal pulmonary artery; b. Moderate intensity and 90% staining of a pulmonary artery with moderate muscular hypertrophy; c. Strong intensity and 100% staining of a pulmonary artery with muscular hypertrophy; d. Strong intensity and 100% staining of a pulmonary artery with severe muscular hypertrophy.

VEGF tissue score was significantly greater in the PH group than control group - saline (p: 0.004), and PH + Bevacizumab group (p: 0.004). Median and ranges were Control Group - saline: 3.9, 3.4 to 4.2; Bevacizumab group: 3.8, 3.5 to 4.2; PH Group: 5.1, 4.8 to 5.3; PH + Bevacizumab group: 4.0, 3.8 to 4.1; p: 0.004 for all groups’ comparison. VEGF tissue scores of the experiment groups are shown in Figure 6. Four different VEGF antibody - stained sections of small pulmonary arteries are shown in Figures 7a–7d.

In the pathological examination, we did not observe emphysema in animals in the experiment groups.

## 4. Discussion

In this experimental study, we investigated the effect of bevacizumab on PH in hypoxia-induced rat model. One of the primary issues in this study was the successful implementation of the hypoxia model. When the normoxia and hypoxia groups were compared, we observed that the animals’ weight measurements in the hypoxia groups on the 5th and 8th days were significantly lower than those in the normoxia groups. RVP and Fulton Index values, which are important indicators of PH, were significantly higher in the hypoxia than the normoxic group. In general, no significant effect of bevacizumab administration was observed in the normoxic group in these measurements. However, in the hypoxic group, there was a significant decrease in RVP, Fulton Index, MT, MT%, VEGF score levels with bevacizumab administration. In our study, findings such as the increase in right ventricular pressures that develop in a short time due to hypoxia and right ventricular hypertrophy are consistent with the previous study results [7,19,27]. Additionally, we thought that the weight loss observed in animals with the application of hypoxia in our study might result from the decrease in oral intake, dehydration, and increase in basal metabolism due to respiratory distress secondary to PH and general condition disorder [15,20,28].

Bevacizumab administration had no statistically significant effect on serum VEGF levels in either control or hypoxia groups. However, some publications in the literature show that serum VEGF levels are higher in humans with PH diagnosis [29] and animals in which experimental PH models were established [13,14] compared to control groups. It was interesting that serum VEGF levels were not affected by bevacizumab administration. We do not have any previously published data on what could be the underlying mechanism for this. However, there can be some possible mechanisms and underlying explanations for this finding. A major explanation may be the statistical power of the study. In experimental animal studies, it is a universal rule that the number of animals per experimental group should be as low as possible for ethical reasons. This ethical rule affects the power of the study, so it becomes challenging to reveal small differences between groups. Again, more than two comparison groups in experimental studies also make it difficult to detect the small differences as statistically significant. In addition, possible experimental errors related to the ELISA - based method we used in serum VEGF measurements may also have affected our results. Thus, it is likely that we may have missed an existing effect due to the methods used in our study.

Hypoxia-induced and monocrotaline - induced models, which are widely used to induce PH in animals, are successful in forming some characteristic pathological changes of PH, such as nonspecific medial and adventitial thickening in pulmonary arteries. Nevertheless, it is known that findings related to chronic, progressive processes of PH such as angio - obliterative plexiform lesions, neointima proliferation, and mononuclear cell infiltration do not develop [27,30,31]. Besides, PH’s pathological and clinical findings may differ in both the distinct species of animals and in different strains of the same species [32,33]. In addition to these differences in animals, PH can also differ between different human groups and races [34,35]. Also, it has been observed that PH, which occurs in hypoxia - induced animal models, is reversible, and pulmonary artery pressures can return to normal with a transition from the hypoxic environment to the normoxic environment [27]. However, PH is generally irreversible in humans. It has been suggested that the hypoxia - induced PH model is closer to milder PH levels in humans and PH secondary to interstitial lung diseases, sleep disorders, chronic obstructive pulmonary disease, and high altitude [27]. When all this information is considered together, we can interpret that although the hypoxia - induced PH model is the classical and most commonly used PH animal model; it cannot fully reflect PH’s mechanism and clinical results in humans. However, we preferred to use this model in our study because it is one of the best-known models. These weaknesses of the model mentioned above should be taken into consideration while evaluating our study findings.

In this study, the effects of bevacizumab, a selective VEGF antibody, on PH were investigated. Since chronic hypoxia is the primary mechanism that triggers VEGF production, the chronic hypoxia model is frequently used in studies to reveal the relationship between PH and VEGF [7,16,29,36,37]. Therefore, we chose to use the same model in our study. VEGF and its tyrosine kinase receptor VEGFR - 2 have an essential role in angiogenesis and endothelial cell protection [38]. It is known that specific VEGFR - 2 inhibitors [39] and VEGFR - 2 monoclonal antibodies [40], and VEGFR - 2 phenotypic knockouts can inhibit neoangiogenesis [38]. Bevacizumab, a VEGF - A monoclonal antibody, is the first antiangiogenic drug approved by the FDA [41] which is used in many cancer types’ treatment schemes, especially colon cancer and non-small cell lung cancer [42]. Case reports and some studies show that bevacizumab can cause PH in individuals with previously normal pulmonary artery pressures by using it in cancer treatment [43,44]. However, this has not been confirmed by studies with large case numbers [45]. Drug’s effect on pulmonary arterial pressures or right ventricular hypertrophy in individuals already diagnosed with pulmonary hypertension.

Previous study findings show that proliferative pulmonary vasculopathy might develop in rats when SU5416 (a VEGF receptor blocker) and hypoxia are applied together. However, unlike bevacizumab, SU5416 blocks VEGF receptors, platelet derived growth factor (PDGF) receptor, c - Kit (stem cell factor receptor), and RET (tyrosine kinase receptor), not VEGF itself, causes emphysema as well as pulmonary hypertension [7]. In contrast to the studies performed with SU5416, we did not observe emphysema in animals in the experimental group in our study. 

It is known that the mechanisms of PH caused by the hypoxia-induced PH model in rodents and the structural changes in tissues are insufficient to reflect many types of PH in humans, especially severe PH types. In this respect, there are problems in generalizing the results of this study to human PH subtypes. However, in this study, it is important that the hypoxia - induced PH model was successfully applied, and significant improvements were observed in many parameters related to PH with bevacizumab administration in hypoxemic rats.

In conclusion, significant decreases in RV pressures, Fulton İndex, medial thickness, medial thickness% scores and tissue VEGF scores detected with bevacizumab application in the hypoxia - induced PH model shows that bevacizumab can affect the pathogenesis and clinical findings in at least some types of PH. It is known that the results of this study cannot completely reflect the human condition due to reasons such as hypoxia - induced animal models are insufficient to reflect many PH subtypes in humans, and bevacizumab was originally produced as a human VEGF antibody. Besides, the role of VEGF in the pathogenesis of PH and the clinical effects of VEGF antibodies and inhibitors in individuals with PH are still unknown. We think that, our study shed a light on the possible effects of a molecule – bevacizumab - used extensively in cancer treatment on the pathogenesis and treatment of PH.

## Informed consent

This manuscript reports the results of experimental investigations conducted with animals. Therefore, informed consent is not applicable.
